# The Practitioner

**Published:** 1873-07

**Authors:** 


***The Practitioner.***

As indicating the growing attention the subject of hygiene is receiving, not only from the public but from the medical profession, we are glad to see that *The Practitioner* (Macmillan & Co., London and New York,) is to be enlarged by the addition of a department devoted to Public Health. The forthcoming number will contain, under this head, articles on Sanitary Organization in England; the Health Aspects of Sewage Irrigation; the Propagation of Typhoid Fever by Milk; International Hygiene in relation to Plague and Cholera.

